# Defending Sperm Function

**DOI:** 10.1371/journal.pgen.1003889

**Published:** 2013-10-24

**Authors:** William Henry Colledge

**Affiliations:** Physiological Laboratory, Department of Physiology, Development and Neuroscience, University of Cambridge, Cambridge, United Kingdom; Stanford University School of Medicine, United States of America

## A New Role for ß-defensins

ß-defensins are a large family of cationic peptides with antimicrobial activities in vitro against Gram-positive and Gram-negative bacteria, fungi, and enveloped viruses [1]. As such, they are thought to be an important component of the innate immune system and are expressed by cells such as phagocytes and epithelia that are involved in host defence against microbial infections. The defensins form pores within the target membranes allowing the movement of small molecules across the plasma membrane and causing decreased bacterial viability. Several ß-defensins are expressed by epithelial cells of the caput epididymis where they may have a role in innate immunity of the urogenital system [2] or protecting sperm from immunorecognition [3]. To date, however, the exact role of ß-defensins in contributing to innate immunity *in vivo* has not been unequivocally established. It is possible that ß-defensins may have other important physiological functions beyond their antimicrobial activities. Indeed, a mutation in the *Defb103* defensin gene causes a dominant black coat colour in dogs [4]. The paper in this issue of *PLOS Genetics* by Yu S. Zhou and colleagues contributes to our understanding of the function of ß-defensins *in vivo* by showing that they also have a role in sperm function [5]. To my knowledge, this is the first unequivocal demonstration of a ß-defensin phenotype in transgenic knock-out mice.

## Events at Fertilization

Spermatozoa show exquisite structural adaptations for their destiny of egg fertilization. The genetic material is tightly compacted into a streamlined head, the mid-piece is packed with mitochondrial powerhouses, and a long tail provides efficient propulsion. The head is covered by the acrosome, a vesicular cap derived from the Golgi that contains enzymes required for fertilization. Freshly ejaculated sperm are motile but not capable of fertilization, as they need to undergo a process called capacitation, which involves biochemical and metabolic changes. These changes include removal of cholesterol from the sperm membranes. Capacitation allows the sperm to bind to the ZP3 protein, a component of the outer zona pellucida layer of the egg. Binding to ZP3 activates T-type and TRPC2 membrane channels that allow Ca^2+^ entry into the sperm to enable acrosome exocytosis. In addition, a cation channel (CatSper) found in the tail region of the sperm that has a promiscuous activation profile can also allow Ca^2+^ entry and may also play a role in facilitating the acrosome reaction. The acrosome reaction is an essential step to allow the sperm to fuse with the plasma membrane of the egg to complete fertilization. Fertilization causes release of Ca^2+^ from intracellular stores in the egg, which induces exit from meiotic arrest and exocytosis of cortical granules to prevent multiple sperm entry.

## ß-defensins Act as a Brake to Sperm Activation

In the article by Zhou et al. [5], the authors have identified that some ß-defensins are essential for normal sperm function and male fertility in the mouse. There are at least 50 ß-defensins genes in the mouse genome with a cluster of 31 located on chromosome 8. Analysis of the function of individual genes in vivo would be a Herculean task and might be compromised by functional redundancy such as in mice deficient for *Defb1*, which show only mild defects [6]. To overcome this problem, the authors took the pragmatic approach of deleting several genes at once. Deletion of a cluster of nine ß-defensin genes (*Defb1*, *Defb50*, *Defb2*, *Defb10*, *Defb9*, *Defb11*, *Defb15*, *Defb35*, and *Defb13*) produced mutant offspring with no obvious gross phenotype or increased inflammatory profiles under normal animal housing conditions. Mutant female mice were fertile, but males were infertile even though histological analysis showed normal spermatogenesis and sperm numbers in the epididymides.

The cause of the infertility was related to multiple defects in the sperm that would compromise their fertilization ability. The sperm showed increased fragility and decreased motility, probably caused by a disrupted microtubule structure in the tail. Around 20% of sperm isolated from the cauda (tail) part of the epididymis showed precocious sperm capacitation, as judged by the appearance of zonadhesin protein on the surface of the sperm. Twenty to twenty-eight percent of the sperm also showed a premature acrosome reaction which normally only occurs when sperm bind to the ZP3 protein. Consistent with the premature acrosome reaction, the mutant sperm showed increased levels of intracellular Ca^2+^ compared to normal sperm.

## Unanswered Questions

The data clearly identify a novel function for ß-defensins in sperm activation and male fertility. It is not yet known, however, whether these ß-defensins play a similar role in humans. Five of the nine ß-defensin genes that were deleted in the mouse have no human orthologues, so it is possible that these effects may be restricted to the mouse. Alternatively, the four genes that are conserved in both humans and mice (*DEFB1/Defb1*, *DEFB106/Defb15*, *DEFB105Defb35*, and *DEFB107/Defb13*) are all expressed in the human testes based on RNAseq expression profiling, so if any of these genes are responsible for the phenotype then they may show functional conservation between humans and mice.

It is also not known what the individual contribution of each of the deleted ß-defensin genes might be towards the phenotype. It is possible that the phenotype might be caused by the loss of only one of the nine ß-defensins genes. Analysis of the expression profile of each of these genes in the epididymis may provide a clue as to which is the most important. For example, the *Defb11* gene is specific to the mouse and shows expression in epididymal epithelia. It might be possible to evaluate the effect of each ß-defensin protein by adding it to mutant sperm and testing whether it can prevent premature capacitation.

**Figure 1:**
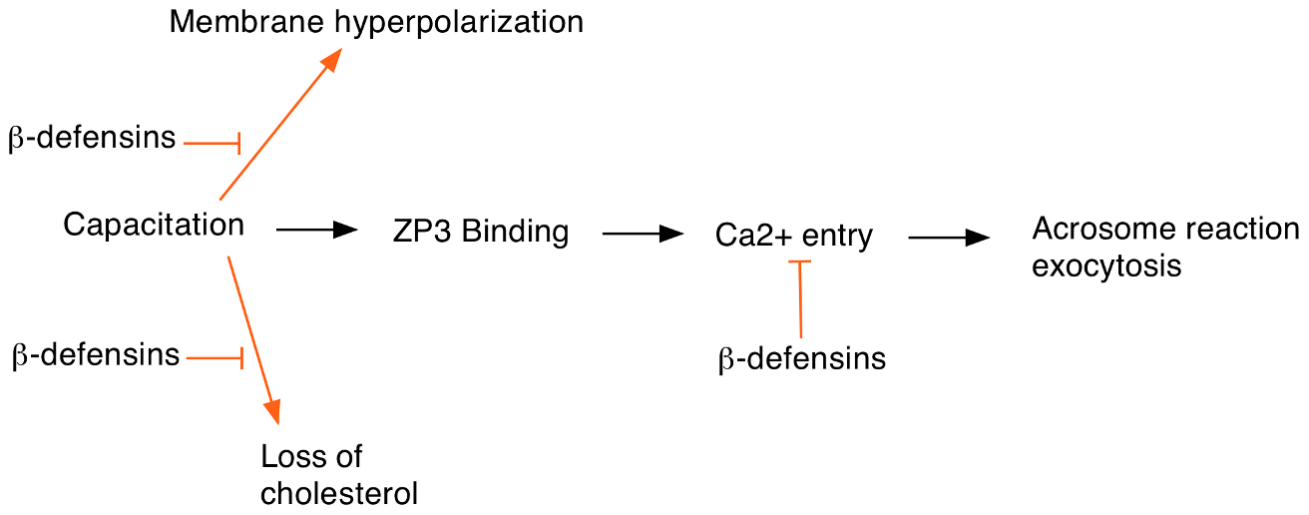
Events at egg fertilization and the possible roles of ß-defensin proteins in regulating this process. Sperm capacitation is required for egg fertilization and involves several cellular changes including loss of cholesterol from the sperm head and increased membrane hyperpolarization. ß-defensin proteins may inhibit these events to help prevent premature sperm capacitation. In addition, ß-defensins might also block Ca^2+^ entry, which is required for the acrosome reaction.

Another important question is to understand the mechanism by which these ß-defensins prevent premature activation of the sperm. The usual sequence of events in the sperm prior to fertilization is shown in Figure 1. Normally Ca^2+^ entry occurs after binding to the egg ZP3 protein although some Ca^2+^ can also enter by promiscuous activation of CatSper channels. The authors speculate that the ß-defensins might inhibit Ca^2+^ channel activity in a way similar to the SPINK3 protein found in seminal fluid. The cationic nature of the ß-defensins will facilitate binding to the sperm plasma membrane where they would be well-placed to interfere with Ca^2+^ channel opening. In addition, the ß-defensins may prevent premature sperm capacitation by inhibiting cholesterol removal from the sperm membranes or preventing hyperpolarization. Biochemical and electrophysiological studies should be able to test these hypotheses and provide a better insight into how ß-defensins modulate sperm activation.
